# First Report of *Trypanosoma cruzi* Infection in Salivary Gland of Bats from the Peruvian Amazon

**DOI:** 10.4269/ajtmh.17-0816

**Published:** 2018-07-16

**Authors:** Fredy E. Villena, Luis A. Gomez-Puerta, Erik J. Jhonston, O. Melisa Del Alcazar, Jorge L. Maguiña, Christian Albujar, V. Alberto Laguna-Torres, Sergio E. Recuenco, Sarah-Blythe Ballard, Julia S. Ampuero

**Affiliations:** 1 U.S. Naval Medical Research Unit No. 6 (NAMRU-6), Lima, Peru;; 2 School of Veterinary Medicine, Universidad Nacional Mayor de San Marcos, Lima, Peru;; 3 Facultad de Medicina de la Universidad Nacional de la Amazonia Peruana, Loreto, Peru;; 4 Departamento de Mastozoología, Museo de Historia Natural, Universidad Nacional Mayor de San Marcos, Lima, Peru; 5 Instituto de Medicina Tropical Daniel A. Carrión, Universidad Nacional Mayor de San Marcos, Lima, Peru;; 6 Departamento de Medicina Preventiva y Salud Pública, Universidad Nacional Mayor de San Marcos, Lima, Peru

## Abstract

In the Americas, 8 million people are infected with Chagas disease, and an additional 90 million people are at risk for infection. Little is known about the role bats play in the sylvatic transmission cycle of *Trypanosoma cruzi*, the parasite causing Chagas disease. Here, we captured bats in the villages of Palmiche, Pachacutec, Nuevo San Martin, and Mayuriaga located in the Datem del Marañon Province in Loreto, Peru. Venous blood samples were collected by cardiac puncture or from the upper extremities, and trypanosomatids were identified by microscopy and molecularly. We collected blood samples from 121 bats on filter paper for molecular studies and 111 slides for microscopic examination of thin and thick blood smears from 16 different bat species. The prevalence of trypanosomatids in all bats species was 34.7% (42/121) and the prevalence of *T. cruzi was* 4.1% (5/121). In hematophagous bat species, the prevalence of trypanosomatids and *T. cruzi* was 36.9% (27/73) and 2.7% (2/73), respectively. In non-hematophagous bats, the prevalences of trypanosomatids and *T. cruzi* were 31.2% (15/48) and 6.2% (3/48), respectively. Also, we confirm the presence of *T. cruzi* in salivary glands of hematophagous bats *Diaemus youngi*. These results suggest a sylvatic cycle of trypanosomatid transmission in which bats may harbor infectious *T. cruzi* parasites that could be transmitted to humans via hematophagous bat bites or salivary contamination by non-hematophagous bats of vegetables consumed by humans.

## INTRODUCTION

American trypanosomiasis, also known as Chagas disease, is an important vector-borne zoonosis caused by the kinetoplastid parasite *Trypanosoma cruzi*.^1,2^ There is evidence that supports the passage of a clade from a common ancestor of *T. cruzi* and *Trypanosoma rangeli* from bats to terrestrial mammals with subsequent diversification and extension. The independent passage of this group gave rise to *T. cruzi* facilitated by the exchange of niches between bats, terrestrial mammals, and invertebrate vectors.^3^ In South America, *T. cruzi* is prevalent in countries between 40° North and 45° South latitudes and is transmitted by insects of the family Reduviidae to mammals of seven different orders, including humans, in both urban and rural settings.^2,4,5^ Chagas disease is endemic in 21 countries in the Americas, infecting 8 million people and posing an additional risk of infection to 90 million people. Because of its widespread distribution in the Americas, the World Health Organization has considered this disease for control and elimination.^6,7^

Studies demonstrate that many mammalian species can serve as hosts in the *T. cruzi* life cycle.^8^ Molecular characterization of the parasite reveals high genetic diversity with two recognized subspecies (*T. c. cruzi* and *T. c. marinkellei*)^9^ and up to seven *T. cruzi* genotypes or discrete typing units (DTUs: TcI to Tc VI and Tcbat).^10,11^

Understanding Chagas disease epidemiology is important for designing effective control strategies. In recent years, *T. cruzi* diagnosis has been greatly improved with the use of molecular biology, which permits clarification of the diversity of *T. cruzi* genotypes and their affinity to specific host mammals.^11^ Through molecular studies, bats have been found to be likely natural hosts of *T. cruzi*^11^ with some species harboring *T. marinkellei* and Tcbat.^11–13^ Although *T. marinkellei* is restricted to bats, evidence that the Tcbat DTU can infect both bats and humans suggests that bats could be part of a sylvatic *T. cruzi* transmission cycle by serving as natural hosts of the parasite.^11–13^ Studies in the coastal and Andean regions of Peru have described the domestic life cycle of *T. cruzi* in guinea pig, dog, goat, and cat hosts, and in *Triatoma infestans* and *Panstrongylus herreri* vectors.^14–18^ In the Peruvian Amazon Basin, the sylvatic life cycle of *T. cruzi* has been described in monkeys (*Saimiri boliviensis*), Andean white-eared opossum (*Didelphis pernigra*), and lesser spear-nosed bat (*Phyllostomus elongatus*) hosts, and in *Panstrongylus geniculatus*, *P. herreri, Panstrongylus chinai, Rhodnius ecuadoriensis*, and *R. pictipes* vectors.^14–20^

In this study, we aimed to assess natural *T. cruzi* infection in bats from different native communities located in the Datem del Marañon Province within the Peruvian Amazon Basin to evaluate the potential role of these species in the transmission of Chagas disease.

## METHODS

### Ethics.

This study was approved by the Institutional Animal Care and Use Committee of the U.S. Naval Medical Research Unit No. 6 (NAMRU-6). Experiments were conducted in compliance with the Animal Welfare Act and in accordance with the principles set forth in the “Guide for the Care and Use of Laboratory Animals,” Institute of Laboratory Animals Resources, National Research Council, National Academy Press, 1996. The Peruvian government agency “Dirección de Gestión Forestal y Fauna Silvestre del Peru (N° 0156)” approved the collection of bat specimens as described. As required by Peruvian regulations, a special entry permit was obtained to enter and perform scientific research in native indigenous territories in the Peruvian Amazon Basin.

### Study sites.

**Figure 1.**
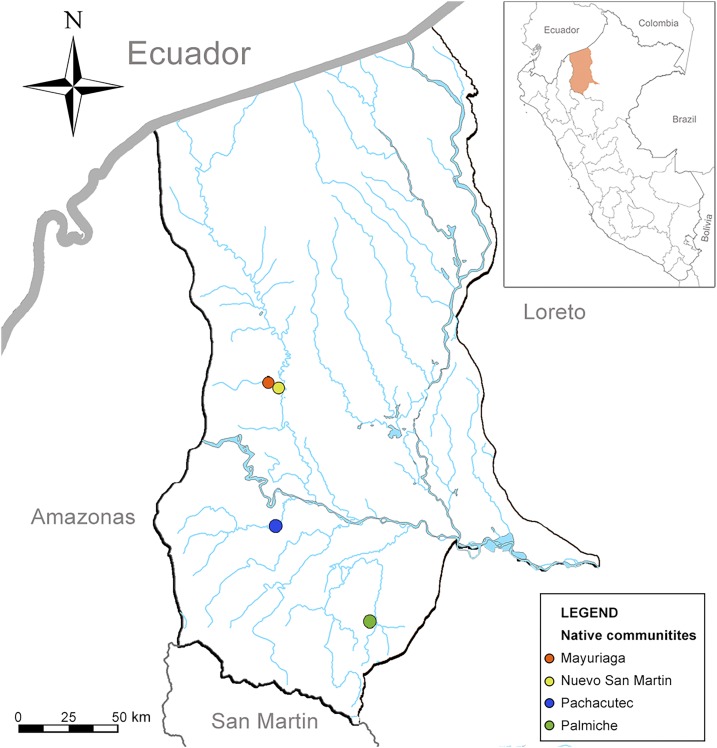
Map of study sites in Loreto-Peru. This figure appears in color at www.ajtmh.org.

The collection of bats was performed in the Loreto Region, Datem del Marañon Province located in the Peruvian Amazon between August 11 and September 3, 2014. Study sites with high and moderate bat bite rates in humans, as reported by the Peruvian General Directorate of Epidemiology, were selected after considering logistical issues, such as accessibility. The selected study sites were Palmiche village (Cahuapanas district), Mayuriaga and Nuevo San Martín villages (Morona district), and Pachacutec village (Barranca district) (Figure 1).

### Specimen collection and identification.

Chiropters were captured in each site during three to four consecutive nights using mist nets around houses and henhouses near villages. Mist nets were numbered consecutively at each site, placed at dusk, and checked at half-hour intervals until midnight. All captured bats were removed from the net and placed inside a canvas bag (one per animal) until processing the following morning in the field laboratory. Once in the field laboratory setting, bats were gently transferred to a Ziploc bag, sedated with isoflurane, euthanized by cervical dislocation, and finally we collected blood samples from the cardiac puncture.

Bats were weighed, measured, sexed, and morphologically identified by genus or species by a mammalogist in the field. Confirmatory identification was subsequently performed based on cranial and dental morphology at the San Marcos Natural History Museum’s Mammalogy Laboratory in Lima, Peru.

Sample size was not calculated because of the surveillance nature of the study. However, we aimed to target vampire bats such as *Desmodus rotundus* in a proportion of 2:1 hematophagous versus non-hematophagous bats at each collection site. Animals captured in excess of this ratio were released.

### Sample collection.

Blood samples were collected from anesthetized animals by cardiac puncture for vampire bats and from the upper extremity veins (small peripheral and median veins) using capillary tubes for insectivorous or frugivorous species. A small amount of blood was also collected on filter paper (Whatman 3MM) for genotype analysis by polymerase chain reaction (PCR) and for microscopic examination of thick and thin blood smears for *T. cruzi.* These samples were transported at room temperature to the NAMRU-6 facilities in Lima and stored at −20°C and 4°C, respectively. Cryovials with blood and tissue samples (kidneys, liver, spleen, lungs, brain, salivary glands, and urine from the bladder) were collected and sent to the NAMRU-6 laboratory in Lima for analysis. All tissue samples were maintained in liquid nitrogen in the field and at −80°C in NAMRU-6, Lima, Peru.

### Microscopic diagnosis of trypanosomatids.

Thick and thin blood smears were stained with Giemsa and examined under an Olympus BX53 microscopy at 100× magnification for the identification of blood-borne parasites. For morphological diagnosis, measurements were performed and pictures were taken using an 18 MP Canon EOS 60D digital camera.

### Molecular diagnosis of trypanosomatids.

DNA was extracted from filter paper imprints, liver, and salivary gland tissues using the QIAamp DNA Mini kit (Qiagen, Hilden, Germany) according to manufacturer’s procedures. A total of ∼30 µL DNA was obtained from each sample and stored at −20°C. A nested PCR was used for amplifying the region specific for 24S α-ribosomal DNA gene of trypanosomatids (∼280 bp) and *T. cruzi* (∼110–130 bp) using the primers D75 (5′-GCA GAT CTT GGT TGG CGT AG-3′) and D76 (5′-GGT TCT CTG TTG CCC CTT TT-3′), and D71 (5′-AAG GTG CGT CGA CAG TGT GG-3′) and D72 (5′-TCA GAA TGG CCG AAC AGT-3′), respectively.^21^ The PCR products were subsequently analyzed by 2% agarose gel electrophoresis and visualized with GelRed stain. The secondary PCR products with the expected size were purified using QIAquick PCR Purification Kit and sequenced using an ABI 3130xL automated sequencer and compared with other sequences of The National Center for Biotechnology Information (NCBI) Genome database (accession numbers: AF288665, M28885, AY367119, AY367115, L14468, GQ303145, AY367116, and RU73612).^22^

## RESULTS

A total of 121 bats belonging to 16 species (families: Vespertilionidae, Phyllostomidae, and Molossidae) were collected during the course of the study (Tables 1 and 2). The family Phyllostomidae predominated with 118 bats identified in the four communities: 36 (30.5%) in Pachacutec, 30 (25.4%) in Mayuriaga, 26 (22.0%) in Palmiche, and 26 (22.0%) in Nuevo San Martin. Only one specimen from the Vespertilionidae family was captured (Pachacutec village) and two from the Molossidae family (one in Mayuriaga and one in Palmiche). Of the 121 bats studied, 54 (44.6%) were males and 67 (55.4%) were females, with a similar proportion in each of the four surveillance sites (Table 2). The distribution of male bats was higher in *Carollia perspicillata* 8/10 (80%), *Diphylla ecaudata* 9/16 (56.2%), and *Diaemus youngi* 4/5 (80%) than other species; the distribution of female bats was greater in *D. rotundus* 37/52 (71.1%), which was the most prevalent species collected, with 52 animals (42.9%) (Table 1).

**Table 1:** Taxonomic identification of hosts and trypanosomatids

									Microscopy		
Characteristics					Sex		PCR		Density of parasites		
Family	Subfamily	Genus	Specie	*n*	Male	Female	Trypanosomatids	*Trypanosoma cruzi*	Mean	Median	Minimum to maximum
Molossidae	Molossinae	*Molossus*	*molossus*	2	1	1	1	–	2	2	2
Vespertilionidae	Vespertilioninae	*Eptesicus*	*brasiliensis*	1	1	–	–	–	–	–	–
Phyllostomidae	Stenodermatinae	*Platyrrhinus*	*brachycephalus*	2	–	2	1	–	1	1	1
	–	*Artibeus*	*planirostris*	15	6	9	3	–	2	1	1–4
	–	–	*lituratus*	4	3	1	1	–	2	2	2
	–	–	*obscurus*	4	3	1	2	–	8	8	8
	–	*Chiroderma*	*villosum*	1	–	1	–	–	–	–	–
	–	*Sturnira*	*lilium*	3	1	2	–	–	–	–	–
	Carolliinae	*Carollia*	*perspicillata*	10	8	2	4	–	4.5	3.5	1–10
	–	–	*castanea*	1	–	1	–	–	–	–	–
	Phyllostominae	*Phyllostomus*	*hastatus*	2	–	2	1	1	1	1	1
	–	*Lophostoma*	*silvicolum*	1	1	–	–	–	–	–	–
	–	*Trachops*	*cirrhosus*	2	2	–	2	2	10.5	1.5	1–20
		Non-hematophagous		48	26	22	15	3	4.2	2	1–20
	Desmodontinae	*Diphylla*	*ecaudata*	16	9	7	7	–	4.3	3	1–14
	–	*Diaemus*	*youngi*	5	4	1	2	1	2	2	1–3
	–	*Desmodus*	*rotundus*	52	15	37	18	1	5.7	3.5	1–25
		Hematophagous		73	28	45	27	2	5.1	3	1–25
Total				121	54	67	42	5	4.8	3	1–25

(–) Represents a value of zero.

**Table 2:** Distribution of chiropters by collection site

				Collection site				
Family	Subfamily	Genus	Specie	Pachacutec	Mayuriaga	Palmiche	Nuevo San Martin	Total
Molossidae	Molossinae	*Molossus*	*molossus*	–	1	1	–	2
Vespertilionidae	Vespertilioninae	*Eptesicus*	*brasiliensis*	1	–	–	–	1
Phyllostomidae	Stenodermatinae	*Platyrrhinus*	*brachycephalus*	–	2	–	–	2
	–	*Artibeus*	*planirostris*	2	–	2	11	15
	–	–	*lituratus*	1	1	1	1	4
	–	–	*obscurus*	–	4	–	–	4
	–	*Chiroderma*	*villosum*	–	1	–	–	1
	–	*Sturnira*	*lilium*	–	–		3	3
	Carolliinae	*Carollia*	*perspicillata*	5	2	3	–	10
	–	–	*castanea*	1	–	–	–	1
	Phyllostominae	*Phyllostomus*	*hastatus*	–	2	–	–	2
	–	*Lophostoma*	*silvicolum*	–	1	–	–	1
	–	*trachops*	*cirrhosus*	2	–	–	–	2
	Desmodontinae	*Diphylla*	*ecaudata*	11	3	2		16
	–	*Diaemus*	*youngi*	2	1	–	2	5
	–	*Desmodus*	*rotundus*	12	13	18	9	52
Sex	–	Males	–	17	16	12	9	54
	–	Females	–	20	15	15	17	67
Age	–	Subadult	–	1	1	–	–	2
	–	Adult	–	36	30	27	26	119
Total				37	31	27	26	121

(–) Represents a value of zero.

The prevalence of *Trypanosoma* sp. and *T. cruzi* by microscopy was 37.8% (42/111) and 1.8% (2/111), respectively. The prevalence of *Trypanosoma* sp. was similar between hematophagous (38.8%, 26/67) and non-hematophagous (36.3%, 16/44) bats. For *T. cruzi,* the prevalence in hematophagous bats was 3.4% (1/26) versus 7.6% (1/16) for non-hematophagous bats.

The mean microscopically measured trypanosome mean body length was 21.2 μm (range: 11.9–37.0 μm; SD = 6.74 μm) and the mean body width was 3.5 μm (range: 2.5–5.1 μm; SD = 0.6 μm) (Table 3).

**Table 3:** Intrinsic characteristics of *Trypanosoma* sp.

Measurements	*n*	Mean	Median	DS	Minimum to maximum	IC 95%
TL	39	21.2	20.5	6.74	11.9–37.0	19.0–23.3
W	38	3.5	3.5	0.63	2.5–5.1	3.3–3.7
NP	37	9.3	8.02	4.16	2.9–18.1	7.9–10.7
NT	37	8.3	7.9	2.96	3.5–15.0	7.3–9.3
LN	39	3.3	3.1	0.93	1.7–6.1	3.0–3.6
LK	39	1	1	0.05	1.0–1.3	0.9–1.0
KN	33	2.4	2	1.94	0.7–9.6	1.7–3.1
KP	35	11	10.3	4.87	0–19.6	9.2–12.6
KT	35	5.6	5.4	3	1.3–13.7	4.5–6.6

TL = total length; W = width; NP = nucleus to the previous part; NT = nucleus to the terminal part; LN = length of the nucleus; LK = length of the kinetoplast; KN = length between the kinetoplast and the nucleus; KP = kinetoplast to the previous part; KT = nucleus to the terminal part.

The overall prevalence by PCR was 34.7% (42/121) for trypanosomatids and 4.1% (5/121) for *T. cruzi*. In hematophagous bats, the PCR prevalence of trypanosomatids and *T. cruzi* were 36.9% (27/73) and 2.7% (2/73), respectively. In non-hematophagous bats, the prevalence of trypanosomatids was 31.2% (15/48) and that of *T. cruzi* was 6.2% (3/48) (Table 1). Compared with females, male bats demonstrated a higher prevalence of *Trypanosoma* sp. and *T. cruzi* with 48.1% (26/54, *P* < 0.005) and 7.4% (4/54, *P* = 0.104) versus 23.8% (16/67) and 1.4% (1/67). Among the bat species identified, the largest crude number of trypanosomatid-positive bats belonged to *D. rotundus* 18/52 (34.6%), followed by *D. ecaudata* 7/16 (43.7%), whereas the highest proportion of trypanosomatid-positive bats belonged to *Trachops cirrhosus* 2/2 (100%), and these two samples were also positive for *T. cruzi.* Also, only the salivary glands of a *D. youngi* specimen were positive by PCR and confirmed by sequencing.

Because the biological material collected from bats did not have sufficient quality for sequencing, we only obtained two sequences in blood samples belonging to two *Trachops cirrhosus*. Phylogenetic analysis of the sequence of the 24 alpha ribosomal gene revealed that these samples had a 98% similarity with the *T. cruzi* lineage TcV (AY367121). However, the sequence obtained from the salivary gland of *D. youngi* had a greater similarity with *T. cruzi* strain Sylvio X10/1 (ADWP02003259).

## DISCUSSION

In Peru, research oriented toward the epidemiology and prevention of Chagas disease in humans and wild mammals is still scarce. Although there are reports of *T. cruzi* in bats from other Latin American countries, to our knowledge, this is the first report of *T. cruzi* in bats from Peru, which hosts a unique ecologic and epidemiologic environment. The presence of *T. cruzi* in bats suggests a bat-associated risk for Chagas disease transmission. Because of their abundance and distribution, bats infected with *T. cruzi* could represent a focus of dissemination of Chagas disease in the Amazonian region of Peru, posing risk to indigenous human populations and mobile populations working and traveling in the Peruvian Amazon Basin.

The detection of trypanosomatids in *D. rotundus*, a hematophagous bat that feeds on blood from pigs, hens, and humans, suggests a pathway for Chagas disease transmission via close contact between the bat body and its fomites with the human victim during blood feeding. This risk is further supported by data showing that the Datem del Marañon Province, where our study was conducted, demonstrates the highest incidence of *D. rotundus* bites in humans in south America reported in the scientific literature.^23,24^

The genus *Trypanosoma* can infect a variety of mammals, including bats, which represent important pathogen reservoirs because of their longevity, variety of shelters, and aggregate population.^11,25^ Moreover, it has been suggested that some trypanosomatids, such as *T. cruzi,* may have adapted first to bat hosts before infecting other terrestrial mammals,^3^ making them likely reservoirs with a potentially active role in *T. cruzi* dissemination.

From all studied communities, 34.7% of collected bats from six different subfamilies (Stenodermatinae, Carolliinae, Phyllostominae, Molossinae, Vespertilioninae, and Desmodontinae) were infected with trypanosomatids with a *T. cruzi* prevalence of 4.1%. This *T. cruzi* prevalence was lower than that of an Argentinian study, which reported an 8% prevalence of *T. cruzi* in bats.^26^ Also, a study from Bolivia reported 15.6% of bats infected with *Trypanosoma* genus but without detection of *T. cruzi*.^27^ In Ecuador, an estimated prevalence of 36.5% for *T. cruzi* and 1.3% for *T. rangeli* was reported.^11^

These differences in *Trypanosoma* prevalence underscore the need for further surveillance to assess changes in parasite distribution across different settings. These epidemiological studies will contribute to integration of opportunities for control and surveillance of Chagas disease and help to characterize the transmission of this parasite in animals and humans within South America.

The relatively uniform prevalence of *T. cruzi* in the four villages in this study (trypanosomatids: Mayuriaga 35.4%, Palmiche 37%, Pachacutec 32.4%, and Nuevo san Martin 34.9%; *T. cruzi*: Mayuriaga 3.23%, Palmiche 3.7%, Pachacutec 5.4%, and Nuevo san Martin 3.8%) suggests that the villages may have common features that contribute to similar parasite distributions. These characteristics that may include weather, geography, diversity of host mammals, and vectors should be further explored.

Our findings show that *T. cruzi* has a wild life cycle in the Peruvian Amazon, involving bats as its host. It is important to note that the occurrence of *T. cruzi* in insectivorous and fruit bats can have public health implications, as the bats could contaminate fruits and vegetables, leading to oral transmission of the Chagas disease as suggested by previous studies.^28,29^

Infection in hematophagous bats (trypanosomatids: 36.9% and *T. cruzi*: 2.7%) could be the result of infection during the bat’s feeding on another infected mammal. This pathway is possible in *Trypanosoma evansi* on *D. rotundus,* which acts as both reservoirs and vectors and that could explain the presence of *T. cruzi* in the salivary glands of *D. youngi*. Another mechanism of infection might occur when regurgitated blood is consumed by juvenile bats from infected adults.^24,26^ In the case of non-hematophagous bats (trypanosomatids: 31.2% and 6.2% *T. cruzi*), infection may be caused by multiple factors such as the ingestion of infected triatomines or other vertebrates, including other bats; vertical transmission from mother to offspring during gestation or breastfeeding; or oral infection. Most of these suggested mechanisms have been confirmed by transmission experiments.^26,27,30–33^ *T. cruzi* and trypanosomatid infection in bats was higher for males (48.1% trypanosomatids, *P* < 0.005 and 7.4% *T. cruzi, P* = 0.104) than for females (23.8% trypanosomatids and 1.4% in *T. cruzi*). This could be due to aggressive behavior during mating season that could lead to an increased risk of injury-associated infections. Like many mammals, male bats migrate to other territories once they reach sexual maturity. This could contribute to disease spread among different colonies, in addition to generating outbreaks in different locations.^34^

For a long time, *T. cruzi* was the only *Trypanosoma* genus involved in human infections. However, recent reports describe human infections caused by other trypanosomatid species that circulate in Latin America such as *T. rangeli* and *Trypanosoma lewisi*.^35,36^ For this reason, epidemiological studies are needed to detect potential vectors and hosts involved in the wild life cycle of the *Trypanosoma* genus.

Between 2006 and 2009, six cases of American trypanosomiasis were identified in the province of Datem del Marañón, which were passively diagnosed through laboratory malaria surveillance. The Mayuriaga village that was evaluated in our study also reported a case of human Chagas disease.^37^ Future studies will assist to develop strategies for prevention, control, and diagnosis of American trypanosomiasis in the Peruvian Amazon Basin which presents a new and a different scenario for Chagas disease in Peru, where populations often live in remote and isolated locations with serious health-care access limitations.

This article is the first exploratory study in Peru that aims to show the presence of potential pathogenic agents for humans in bats. Therefore, additional studies are needed to incriminate bats in the transmission of *T. cruzi* to humans.

## LIMITATIONS

As it was not an exclusive study for *T. cruzi*, we could not collect the data needed to confirm direct transmission of *T. cruzi* from bats to humans. Also, the blood samples in filter paper collected from bats did not have sufficient quality for sequencing of multiple genes because of their storage and transport at room temperature.
